# Language lateralization of hearing native signers: A functional transcranial Doppler sonography (fTCD) study of speech and sign production

**DOI:** 10.1016/j.bandl.2015.10.006

**Published:** 2015-11-19

**Authors:** Eva Gutierrez-Sigut, Richard Daws, Heather Payne, Jonathan Blott, Chloë Marshall, Mairéad MacSweeney

**Affiliations:** a Deafness, Cognition & Language Research Centre, University College London, United Kingdom; b Institute of Cognitive Neuroscience, University College London, United Kingdom; c UCL Institute of Education, University College London, United Kingdom

**Keywords:** fTCD, Language lateralization, Language production, Sign language, Phonological fluency, Semantic fluency, Hearing native signers

## Abstract

Neuroimaging studies suggest greater involvement of the left parietal lobe in sign language compared to speech production. This stronger activation might be linked to the specific demands of sign encoding and proprioceptive monitoring.

In Experiment 1 we investigate hemispheric lateralization during sign and speech generation in hearing native users of English and British Sign Language (BSL). Participants exhibited stronger lateralization during BSL than English production. In Experiment 2 we investigated whether this increased lateralization index could be due exclusively to the higher motoric demands of sign production. Sign naïve participants performed a phonological fluency task in English and a non-sign repetition task. Participants were left lateralized in the phonological fluency task but there was no consistent pattern of lateralization for the non-sign repetition in these hearing non-signers.

The current data demonstrate stronger left hemisphere lateralization for producing signs than speech, which was not primarily driven by motoric articulatory demands.

## 1 Introduction

The left hemisphere plays a critical role in language processing in the majority of the population (Hellige, 1993). Large scale studies of language lateralization show that 82—96% of right handed participants primarily use their left hemisphere for spoken language processing (Knecht, Deppe, et al., 2000; Knecht, Drager, et al., 2000; Springer et al., 1999). Similarly, lesion and neuroimaging studies have shown that signed languages appear to rely on a left lateralized network for both comprehension and production (Bellugi, Klima, & Poizner, 1988; Corina, 1999; Corina et al., 1999; Damasio, Bellugi, Damasio, Poizner, & Van Gilder, 1986; MacSweeney, Capek, Campbell, & Woll, 2008; MacSweeney, Waters, Brammer, Woll, & Goswami, 2008). Although neural networks supporting speech and sign processing are very similar, they are not identical. In addition to analogous activation in the classical left perisylvian areas for sign and speech, previous neuroimaging studies have also identified the left parietal lobe as having a greater role in signed than spoken language processing (for a review see Corina, Lawyer, & Cates, 2012; MacSweeney, Capek, et al., 2008; MacSweeney, Waters, et al., 2008). Studies of sign production in particular have highlighted an important role for the left parietal lobe (Braun, Guillemin, Hosey, & Varga, 2001; Corina, San Jose-Robertson, Guillemin, High, & Braun, 2003; Emmorey, Mehta, & Grabowski, 2007).

Sign and speech production differ dramatically in terms of articulatory and motoric demands. Overt generation of words requires rapidly changing movements of the vocal tract and the mouth, which are non-lateralized anatomic structures. In contrast, sign articulation demands precise movements of the face, torso and, crucially, the arms and hands. Although sign production involves both hands, signers are dominant in the use of one hand or the other (Vaid, Bellugi, & Poizner, 1989). Different signs can require different degrees of involvement of the hands. Three simple categories can be considered: (1) one-handed signs, performed by the signer’s ‘dominant’ hand, (2) two handed signs in which the dominant hand carries out most of the movement required for the sign, (3) two-handed signs with equal movement of both articulators (for a linguistic description of these types of signs see Battison, 1978). This differential use of the hands has direct implications for brain activity since movements of the hands are associated with the contralateral brain hemisphere. Therefore, in right hand-dominant signers the left motor cortex would be engaged more than the right during sign production and vice versa for left hand dominant signers.

Another area of difference between sign and speech is in their use of self-monitoring mechanisms. Speech is directly audible to the speaker. In contrast, the signer does not have complete perceptual feedback of her own signing. Even when she can see her hands moving in space, her point of view is different to that of regular sign perception. This raises the likelihood that sign production relies more on proprioceptive and somatosensory feedback than speech (see e.g. Emmorey, McCullough, Mehta, & Grabowski, 2014). The need to keep track of the position and precise movements of the hands may also influence hemispheric lateralization observed in signers.

A number of previous studies, that have used fMRI and PET to examine the neural systems supporting sign and speech production, have identified the left parietal lobe as playing a greater role in sign than speech production (e.g. Braun et al., 2001; Emmorey et al., 2007). For example, Emmorey et al. (2014) used H_2_^15^O-PET to directly contrast ASL and English production during picture naming in hearing native signers, without removing low-level motoric effects. Sign production led to greater parietal activation than English, especially in the left hemisphere. This greater activation was attributed to somatosensory and proprioceptive feedback, the voluntary production of motor movements, and sensory motor integration necessary for phonological encoding of signs.

However, technical limitations of neuroimaging techniques, such as the need to minimize movement, have meant that previous studies of sign language production have required covert speech generation or sign whispering, a form of signing with a displaced location and a reduced amount of movement (Emmorey et al., 2007, 2014). Furthermore, in order to minimize movement in the scanner, many previous studies have only examined the production of one-handed signs (Corina et al., 2003; Emmorey et al., 2007) or have not considered the amount of movement actually executed by each hand (Braun et al., 2001). These factors might influence the patterns or the strength of motor cortex activation differently than when producing signs in more naturalistic conditions. Brain activity linked to somatosensory feedback is likely to differ when the production is limited by the technique’s technical restrictions.

The current study uses functional transcranial Doppler sonography (fTCD) to investigate hemispheric lateralization during natural, non-whispered BSL and English production. fTCD measures event-related changes in blood flow velocity to the middle cerebral arteries bilaterally. fTCD is a non-invasive, fast and safe technique for establishing hemispheric dominance during cognitive tasks (Aaslid, 1987; Bishop, Watt, & Papadatou-Pastou, 2009; Deppe, Knecht, Lohmann, & Ringelstein, 2004) which shows high concordance with functional magnetic resonance imaging (fMRI; Deppe et al., 2000; Somers et al., 2011) and the Wada technique (Knake et al., 2003; Knecht, Deppe, Ebner, et al., 1998; Knecht, Deppe, Ringelstein, et al., 1998). fTCD is considered an excellent technique for measuring hemispheric dominance for language production, especially in children and populations for whom MRI is not an option (e.g. cochlear-implanted participants). One of the reasons in favour of using fTCD is that the signal is reasonably robust to movement (e.g. Gutierrez-Sigut, Payne, & MacSweeney, 2015; Knecht, Deppe, & Ringelstein, 1999), meaning that neither the quality of the signal nor the patterns of lateralization seem to be affected by a participant’s natural speech articulation (Badcock, Nye, & Bishop, 2012; Bishop et al., 2009; Gutierrez-Sigut, Payne, et al., 2015). To address this issue we have previously contrasted covert and overt speech production directly (Gutierrez-Sigut, Payne, et al., 2015). We reported no differences between covert and overt speech in the number of epochs with movement artefacts, in the strength of lateralization or in the percentage of individuals categorised as left lateralized.

Unlike speech movements, however, sign language production requires constant movement of the arms and hands. Understanding how this type of movement affects the strength of lateralization measured with fTCD becomes especially relevant since, as discussed above, these movements tend to be asymmetrical. Here we investigate whether the strength of lateralization is similar for natural speech and sign production in two different overt language generation tasks: phonological and semantic fluency.

The phonological fluency task is considered the gold standard task for assessing language lateralization with fTCD in adult speakers. In this task the participant is presented with a series of letters one at a time and asked to generate as many words beginning with the letter as possible within a fixed time window. While phonological fluency is a reliable and remarkably consistent task, the examination of other language domains provides a more complete pattern of hemispheric dominance for language.

Gutierrez-Sigut, Payne, et al. (2015) used fTCD to study lateralization in native English speakers. In addition to a standard phonological fluency task, participants performed a semantic fluency task. In this task participants were given a semantic category (e.g. animals) and asked to produce as many words from this category as possible in the given time. Results showed a similar percentage of left lateralized participants (phonological: 77% vs. semantic: 82%) as well as no significant differences in strength of laterality between phonological and semantic tasks. In another of our studies we asked participants to make rhyme judgments (Payne, Gutierrez-Sigut, Subik, Woll, & MacSweeney, 2015). Only 66% of participants in the fast version of this task were classified as showing left hemisphere lateralization. This suggests that the language demands of the task used can, perhaps not surprisingly, influence the strength of the lateralization. This result suggests that, in order to further our understanding of the contribution of fTCD to the field, it is critical to take a multidimensional approach and examine lateralization for language across a range of tasks (Payne et al., 2015).

Comparing lateralization across language subdomains can allow further insights into the question of what aspects of language processing are influenced by modality and which are not. It can also be useful for assessing consistency of lateralization patterns within individuals. In a recent study, Marshall, Rowley, and Atkinson (2014) used phonological and semantic fluency tasks to study the organization of the lexicon in deaf BSL users. Results from the semantic fluency task showed expected similarities with spoken such as an equivalent number of overall responses. Responses to phonological categories, however, were remarkably less productive in the signers than they typically are in speakers. Moreover, analysis of the types of clustering within tasks/categories revealed a close relationship between semantics and phonology in the signs generated. Similarly, in a study of ASL comprehension, Gutierrez, Williams, Grosvald, and Corina (2012) found electrophysiological evidence of an interplay between phonological form and meaning in native signers. ERP responses to sentences, in which semantic expectancy and phonological form were systematically manipulated, showed a similar early onset N400 for semantically related and phonologically related signs. This result is interpreted as evidence that semantic and phonological properties are accessed early in ASL comprehension and incur similar on-line processing costs.

In the present study we compare within-participant responses, to phonological and semantic fluency tasks in both BSL and English. Hearing native signers were tested. These individuals were born to Deaf parents from whom they learnt BSL and they also grew up learning spoken English from the hearing community around them. This allows us to directly contrast, within individuals, the strength of lateralization in both languages.

In summary, there is accruing evidence of increased left parietal involvement in sign compared to speech production linked with proprioceptive monitoring and the special nature of phonological encoding of signs. Here we investigate lateralization strength during both BSL and English semantic and phonological fluency tasks. First, we examine the extent to which fTCD measures are robust to overt production of natural (non-whispered) signs. We assess this by examining the number of epochs rejected due to poor data quality in both languages and by examining the reliability of fTCD measures. Second, we predict that hearing native signers will produce more items (either signs or words) in the semantic than in the phonological tasks in both languages. Third, we expect similar levels of lateralization between phonological and semantic tasks in English. Whether the same would be true for BSL is an open question. Finally, our primary interest is in the strength of lateralization across sign and speech production. If the generation of signs leads to an increased left hemisphere involvement we would expect to observe a stronger lateralization for BSL than English. Furthermore, if the strength of lateralization, measured with fTCD, is largely driven by the actual hand and arm movement, we would expect a correlation between the amount of movement and laterality index in signers.

## 2 Experiment 1

### 2.1 Methods

#### 2.1.1 Design

We used a 2 (*Language*: English vs. BSL) × 2 (*Task*: phonological vs. semantic) design. The resulting four conditions were presented in separate blocks, the order of which was counterbalanced across participants: English-phonological, BSL-phonological, English-semantic and BSL-semantic.

#### 2.1.2 Participants

A total of 16 participants (14 female) were selected using the Deafness Cognition and Language research centre (DCAL) participant database. The mean age of participants was 34 years (range 19–49, SD = 8.8). All participants were hearing BSL native signers, with at least one of their parents being a deaf signer. Mean self-rated BSL proficiency, in a scale from 1 (poor) to 7 (excellent), was 5.8 (range 4–7; SD = 1.2). Eight participants were trained interpreters. No participants reported a history of neurological disorders or language related problems. Participants were all right handed as assessed by an abridged version of the Edinburgh Handedness Inventory (Oldfield, 1971). The questionnaire was composed of ten questions about handedness and four questions related to footedness for regular activities. Participants were all right hand dominant for BSL signing, fingerspelling and counting.

#### 2.1.3 Stimuli

##### 2.1.3.1 English phonological fluency

10 letters were chosen which have been reliably used in previous phonological fluency studies (see e.g. Gutierrez-Sigut, Payne, et al., 2015): A, B, C, F, H, M, O, S, T, W. Each letter was presented twice. Thus, each condition consisted of 20 trials that were presented in a pseudo-randomized order to ensure that all 10 letters had been presented once before they were repeated.

##### 2.1.3.2 English semantic fluency

10 semantic categories were chosen: Farm Animals, Zoo Animals, Vegetables, Fruits, Drinks, Colours, Sports, Pets, Tools and Transport. These categories were repeated twice, resulting in 20 trials per block that were presented in a pseudo-randomized order (as above).

##### 2.1.3.3 BSL phonological fluency

**Fig. 1:**
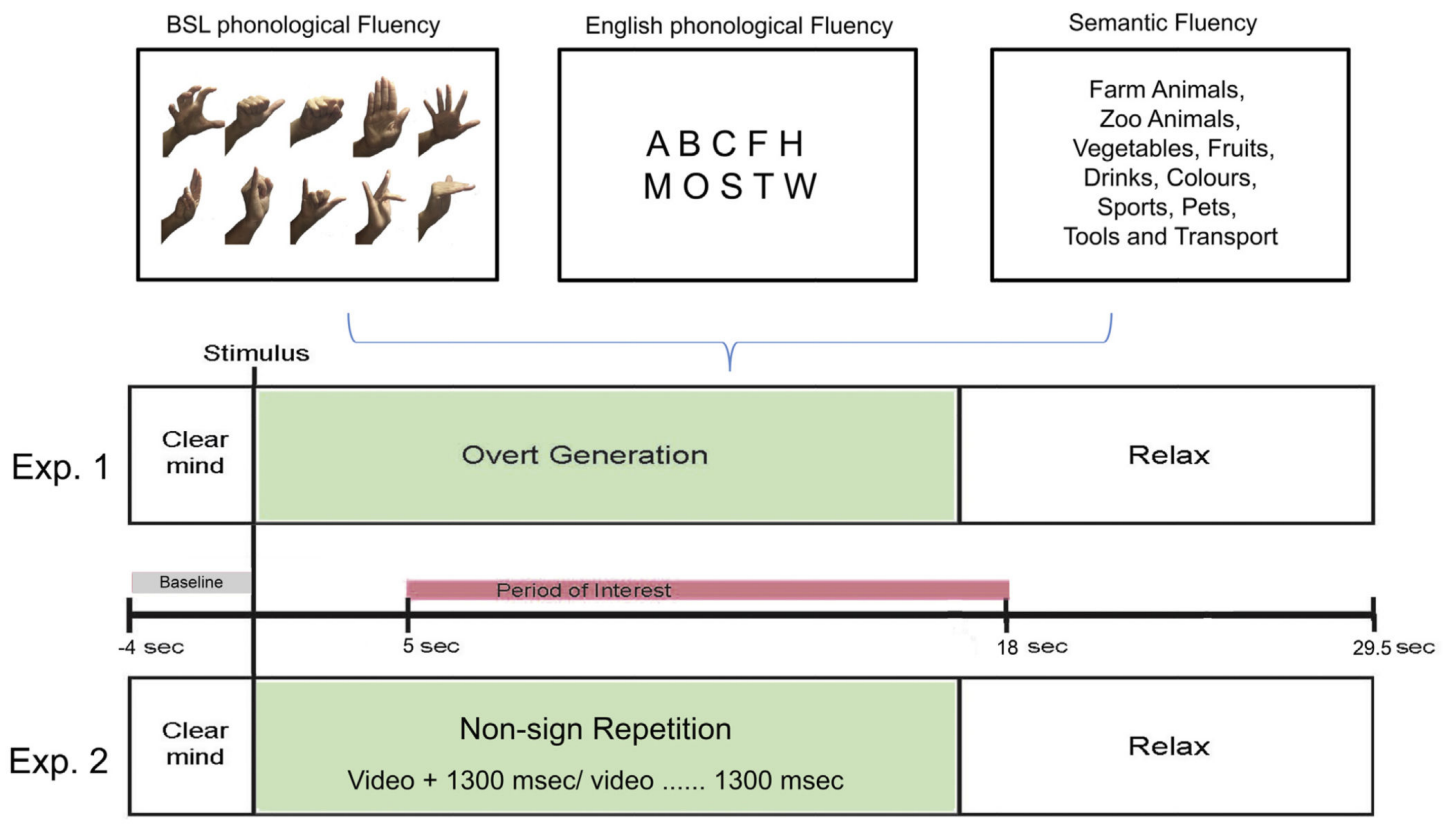
Schematic diagram of experimental material (top panel) and timing of events in Experiment 1 (central panel) and timing of events in Experiment 2 (bottom panel).

10 BSL handshapes were chosen (see Fig. 1, top panel) from the most productive BSL handshapes in the dictionary of British Sign Language (Durham University, 1992). As with the letters, each handshape was presented twice. Thus, each condition consisted of 20 trials that were presented in a pseudo-randomized order.

##### 2.1.3.4 BSL semantic fluency

The semantic categories were the same as those chosen for the English task. As above, each category was repeated twice, resulting in 20 trials per block that were presented in a pseudo-randomized order.

#### 2.1.4 Procedure

Ethical approval for the study was obtained from the UCL Research Ethics Committee. All participants gave written informed consent prior to the study. The whole session, including set up time, lasted approximately 2 h. Each block was preceded by two practice trials showing categories, letters or handshapes that were not used in the experimental blocks.

##### 2.1.4.1 English blocks

Each trial began with a four second preparation period during which ‘clear your mind’ was displayed on the screen and participants were instructed to focus on the screen (see Fig. 1). The cue, either a single letter or a semantic category, was then displayed for 17 s. Participants were asked to overtly generate as many words as possible beginning with the letter (phonological condition)/belonging to the category (semantic condition) displayed on the screen. A ‘relax’ prompt then appeared for 12.5 s. Participants were instructed to use the ‘relax’ period to imagine a peaceful scene. The overall trial duration was 33.5 s, which is shorter than many previous studies of word generation (see e.g. Knecht, Deppe, Ringelstein, et al., 1998). Nonetheless, we have previously obtained reliable fTCD measures using this task with a 30 s epoch and comparable generation and relax periods (Gutierrez-Sigut, Payne, et al., 2015).

Stimuli were presented using Cogent toolbox (www.vislab.ucl.ac.uk/cogent) for MATLAB (Mathworks Inc., Sherborn, MA, USA). Triggers time-locked to the onset of the stimulus were sent from the presentation PC to the Doppler-Box set-up.

##### 2.1.4.2 BSL blocks

The BSL blocks proceeded in exactly the same way as the English ones, except that all prompts and cues were given in BSL. ‘Clear your mind’ and ‘relax’ messages, as well as the semantic categories were presented as video clips. After the sign for the semantic category cue was completed the last frame remained on the screen for the whole generation period. The phonological cues (handshapes) were static images.

#### 2.1.5 fTCD recording and processing

Changes in blood flow velocity in the left and right MCAs were measured using a Doppler ultrasonography device (DWL DopplerBox: manufactured by DWL Elektronische Systeme, Singen, Germany). Two 2-MHz transducer probes were mounted on a flexible headset and placed at each temporal skull window.

Data analysis was carried out with dopOSCCI, a custom MATLAB (Mathworks Inc., Sherborn, AM, USA) program written for analysing fTCD group data (Badcock, Holt, Holden, & Bishop, 2012). Analysis involved down-sampling of the data from 100 to 25 Hz, normalization of left and right channel values on an epoch by epoch basis, heart cycle integration and artefact rejection. Epochs containing blood flow velocities less than 70% or greater than 120% of the average blood flow velocity were excluded from the analyses. Epochs were segmented from −4 to 29.5 s (33.5 s long) relative to stimulus presentation. All data points were baseline corrected by subtracting the averaged blood flow velocity during a period of inactivity −4 to 0 s prior to stimulus onset. The period of interest (POI) was set from 5 to 18 s after stimulus onset. To ensure that blood flow for the baseline period was always calculated from resting level, the first trial of the block was not included in the analyses. This resulted in 19 analysed trials per block. Laterality indices (LIs) were calculated for each participant separately, for each of the four conditions (Badcock, Holt, et al., 2012; Badcock, Nye, et al., 2012; Deppe, Knecht, Henningsen, & Ringelstein, 1997). For each participant the maximum peak left-right difference within the POI was identified. The two-second measurement window was centred on this maximum. The LI was defined as the average of the left minus right differences within this two second window. A positive LI is indicative of left lateralization and a negative LI of right lateralization.

One-sample *t*-tests were used to assess whether the LI value was significantly left or right lateralized for each participant in each condition. When the one-sample *t*-test did not reach significance, participants were considered as ‘low lateralized’ for that condition (also referred to as ‘bilateral lateralization’ e.g. Badcock, Holt, et al., 2012; Badcock, Nye, et al., 2012; Bishop et al., 2009).

#### 2.1.6 Behavioural responses and movement coding

Participants’ behavioural responses were monitored on-line and videos were recorded for scoring offline. In the English phonological fluency conditions, items were accepted if they began with the target letter or letter ‘sound’ (e.g. phone for /f/ was allowed). In the BSL phonological fluency task items were accepted if they were a real sign that can be articulated with the prompted handshape. Occasionally participants produced a non-sign, that is they used the cued handshape to produce a sign that should be articulated with a similar handshape. These items were not scored as correct. In the semantic conditions, all items semantically linked to the category were permitted.

In order to explore the effect on the TCD signal of arm and hand movement during sign generation, participants movements during the 17 s generation period were coded by a deaf BSL signer. Three categories were used for this coding: (1) the participant made a one-handed sign moving only the right hand, (2) the participant made a two-handed sign in which the right hand was dominant^1^ and (3) the participant made a two-handed sign in which both hands move symmetrically. All linguistic and non-linguistic movements were coded as one handed, right hand dominant or two hands symmetrical. Instances of left hand alone movement were extremely scarce and therefore not coded.

### 2.2 Results

#### 2.2.1 fTCD data quality and reliability

In order to investigate whether overt signing led to more movement artefacts in the fTCD data than overt speech we analysed the number of epochs remaining for each participant after artefact rejection (see Methods). A repeated measures ANOVA revealed no differences in the number of epochs accepted between languages [*F*(1,15) < 1], or tasks [*F*(1,15) = 1.7, MSE = 1.09, *p* > .2] and there was no significant interaction [*F*(1,15) < 1]. The average number of accepted epochs for each condition was 18 (min = 12, max = 19, SD = 1.2).

To assess the internal reliability of the data, Pearson’s correlations between the LIs of the even and odd epochs were conducted on each condition separately. Good reliability was found for the fTCD data: odd and even epochs were correlated within the English-phonological (*r* = .817, *p* < .001), BSL-phonological (*r* = .823, *p* < .001), English-semantic (*r* = .637, *p* < .01) and BSL-semantic (*r* = .858, *p* < .001).

In order to assess the consistency of LIs across conditions we examined the correlations between LIs. Positive correlations were found between the English phonology and BSL phonology tasks (*r* = .693, *p* < .01) and between the English semantic and BSL semantic tasks (*r* = .556, *p* < .05). Within languages, LIs on both tasks also correlated or approached the level of significance: BSL phonology and BSL semantic (*r* = .757, *p* < .001) and English phonology and English semantic (*r* = .49, *p* = .054).

#### 2.2.2 Mean LI and percentage of subjects left lateralized

**Fig. 2:**
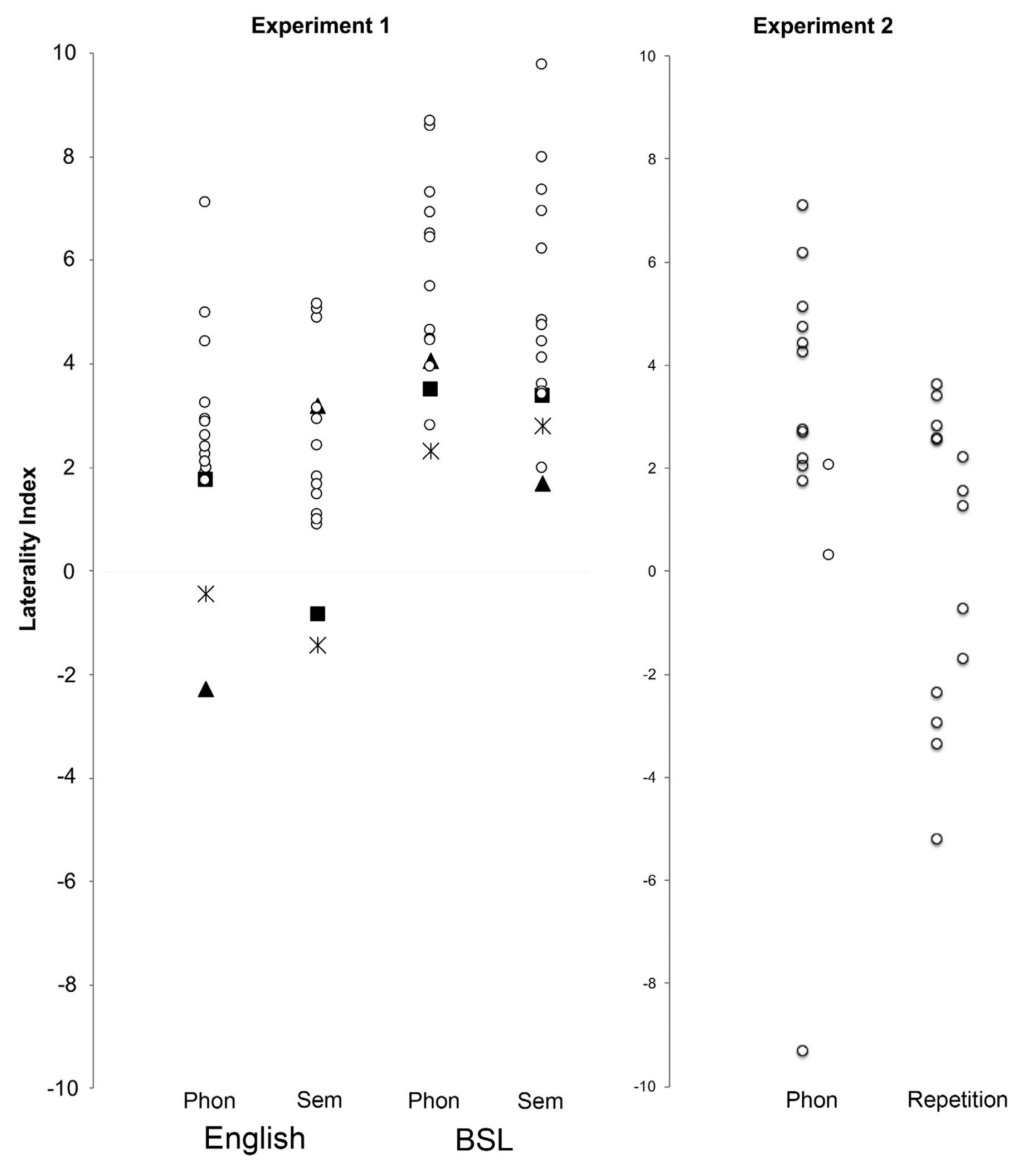
Individual LI scatterplots for each condition. The left panel shows Experiment 1. The LIs for atypical individuals in any of the four conditions are shape coded, each shape consistently codes each of these three participants across conditions. The right panel shows Experiment 2. Individual LI for phonological fluency are shown on the left and for non-sign repetition on the right of the scatterplot.

At the group level, each of the four conditions was significantly left lateralized (see Table 1 and Fig. 2). However, not all participants showed this pattern. Table 1 shows the number of participants who showed left lateralization, low laterality (not significantly different to zero) or were right lateralized (negative LI, significantly different to zero) in each condition. This variability is also clear in Fig. 2. The three participants who had a negative LI in any of the four conditions are shape coded for ease of tracking across conditions. Detailed visual inspection of the data from these participants did not show increased artefacts or signal noise.

#### 2.2.3 LI differences between conditions

**Fig. 3:**
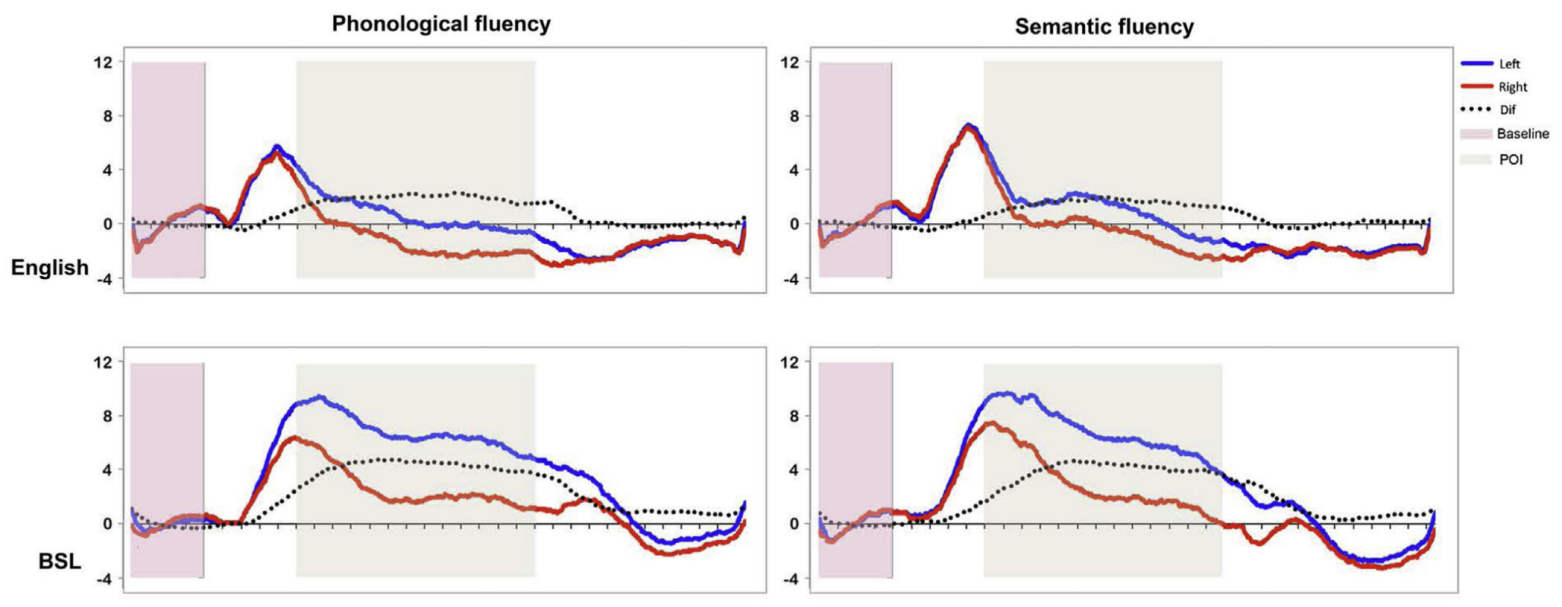
Average of participants’ baseline-corrected cerebral blood flow velocity for the left (blue) and right (red) channels as well as the difference (left minus right; black dotted line) for phonological (right) and semantic fluency (left) in English (top) and BSL (bottom). The beige selection depicts the period of interest within which the lateralization indices (LIs) were calculated from the individuals’ maximum left–right difference. (For interpretation of the references to colour in this figure legend, the reader is referred to the web version of this article.)

A repeated measures ANOVA showed a main effect of *Language* [*F*(1,15) = 89.3, MSE = 1.29, *p* < .0001, $\eta_{\text{P}}^{2}$ = .856]: LIs for BSL were stronger than for English (4.997 vs. 2.35). The main effect of *Task* [*F*(1,15) = 1.76, MSE = 1.21, *p* > .1, $\eta_{\text{P}}^{2}$ = .105] as well as the interaction [*F* < 1, $\eta_{\text{P}}^{2}$ = .003] were not significant. Fig. 3 shows cerebral blood flow velocities for the left and right channels time-locked to the beginning of the active period.

#### 2.2.4 Relationship between LI and number of items generated

##### 2.2.4.1 Behavioural data: number of items produced during BSL and English fluency tasks

Table 2 shows the average number of items (words or signs) reported for each trial in each of the four conditions for the 16 participants. A repeated measures ANOVA on the number of correctly produced items showed that the main effect of language was significant [*F*(1,15) = 53.7, MSE = 1.59, *p* < .0001, $\eta_{\text{P}}^{2}$ = .78]. The main effect of Task was significant [*F*(1,15) = 71.84, MSE = 1.2, *p* < .0001, $\eta_{\text{P}}^{2}$ = .83]. The interaction was also significant [*F*(1,15) = 16.97, MSE = .727, *p* < .001, $\eta_{\text{P}}^{2}$ = .53]. Pairwise comparisons showed that participants produced more items in the semantic than in the phonological task in English (mean of 8.7 vs, 7.3 [*F*(1,15) = 19.82, *p* < .0001, $\eta_{\text{P}}^{2}$ = .569]) and BSL (mean of 7.3 vs. 4.1 [*F*(1,15) = 75.4, *p* < .0001, $\eta_{\text{P}}^{2}$ = .834]). Moreover, participants produced more items in English than BSL in both tasks: phonological (mean of 7.3 vs. 4.1 [*F*(1,15) = 139.5, *p* < .0001, $\eta_{\text{P}}^{2}$ = .903]) and semantic (mean of 8.7 vs. 7.3 [*F*(1,15) = 9.45, *p* < .01, $\eta_{\text{P}}^{2}$ = .387]).

##### 2.2.4.2 Correlations between number of words produced and LIs

No significant correlations were found between strength of LI and number of items produced in any condition: English phonological (*r* = −.084, *p* > .5); English semantic (*r* = −.102, *p* > .5); BSL phonological (*r* = .036, *p* > .8); BSL semantic (*r* = .38, *p* > .1).

This lack of relationship was not due to inclusion of the three participants that had LI values lower than 0 in any of the conditions, and therefore a right hemisphere bias. When these participants were excluded, no significant relationships between LI and number of items produced were found: English phonological (*r* = −.202, *p* > .5); English semantic (*r* = −.375, *p* > .2); BSL phonological (*r* = −.025, *p* > .9); BSL semantic (*r* = .035, *p* > .9).

#### 2.2.5 Relationship between LI and sign type

##### 2.2.5.1 Behavioural data: handedness of signs produced during BSL fluency tasks

To examine the relationship between hand movement and LI we undertook detailed analysis of the BSL signs produced (see methods). A repeated measures ANOVA of task (phonological and semantic) and sign type (right hand only, right hand dominant and both hands symmetrical) showed a main effect of sign type [*F*(2,30) = 71.78, MSE = 2,046,578, *p* < .0001, $\eta_{\text{P}}^{2}$ = .827]. Pairwise comparisons showed that more time during each trial was spent producing right hand only movements than two-handed right hand dominant movements (mean of 6.14 s per trial vs. 2.37 s, SD of 1.9 and .99, minimum of 3.3 and .8, maximum of 10.4 and 4 respectively; [*p* < .0001]) and both hands symmetrical movements (mean of 6.14 s per trial vs. 2.84 s, SD of 1.9 and .91, minimum of 3.3 and 1.5, maximum of 10.4 and 4.4 respectively; [*p* < .0001]). There were no differences in the amount of time spent producing right hand dominant two handed movements and both hands symmetrical movements (*p* > .1). There was no main effect of *Task* [*F*(1,15) = 2.19, MSE = 1,031,174, *p* > .1, $\eta_{\text{P}}^{2}$ = .127] or interaction [*F* < 1, $\eta_{\text{P}}^{2}$ = .026].

##### 2.2.5.2 Correlations with LI

There was a moderate significant correlation between LI during the BSL phonological task and right hand only movements (*r* = .5, *p* < .05) but not with right hand dominant (*r* = −.14, *p* > .1) or both hands symmetrical movements (*r* = −.03, *p* > .9). For BSL semantic generation there was no significant correlation between LI with any of the types of movement: right hand only (*r* = −.05, *p* > .8), right hand dominant (*r* = −.21, *p* > .4) or both hands symmetrical movement (*r* = −.3, *p* > .1).

As mentioned above, two-handed signs in which the right hand is dominant are composed primarily of movement of the right hand. In order to explore more thoroughly the effects of dominant hand movement, we combined the right hand only and right hand dominant conditions. For phonological generation there was a moderate significant correlation of right hand movement and LI (*r* = .54, *p* < .05). There was no correlation for semantic generation (*r* = .088, *p* > .7). Fig. 5 shows the relationships of the different types of hand movement with LI in both the phonological and semantic tasks.

### 2.3 Discussion

Our main finding was that of significantly stronger LIs for BSL than English. This increased laterality for BSL was observed for both semantic and phonological generation. The movement analyses demonstrated a moderate correlation between LI and right hand movement in the phonological generation task. This does not allow us to rule out the possibility of some contribution of motoric brain activation to strength of lateralization. This finding encouraged a further study of the role of movement during sign production in Experiment 2.

## 3 Experiment 2

We investigated the role of movement during sign production further by comparing lateralization patterns during English phonological fluency and a non-sign repetition task in which hearing non-signers were asked to repeat non-signs (structured hand movements that have no semantic content). We hypothesized that if hand movement by itself elicits strong LIs then the majority of participants (all right-handed non-signers) would show left hemisphere lateralization during non-sign repetition. Conversely, if the strong lateralization for BSL production observed in Experiment 1 in signers is not primarily driven by motor activity but by linguistic processing we will find stronger LIs for English phonological fluency than for non-sign repetition.

### 3.1 Methods

#### 3.1.1 Design

Participants performed two tasks, overt English phonological fluency and non-sign repetition. The tasks were presented in separate blocks, the order of which was counterbalanced across participants.

#### 3.1.2 Participants

A total of 16 participants (4 male) were recruited from undergraduate courses at UCL and also from a volunteer database. The mean age of participants was 23 (range 19–34) and all were English native speakers. All participants were hearing and did not have any previous knowledge of BSL. No participants reported a history of neurological disorders or language related problems. Participants were all right handed as assessed by an abridged version of the Edinburgh Handedness Inventory (Oldfield, 1971).

#### 3.1.3 Stimuli

##### 3.1.3.1 Phonological fluency in English

The same 10 letters as in Experiment 1 were used. Four of the letters were presented twice, and letters to be repeated were chosen randomly for each participant. Each block consisted of 14^2^ trials which were presented in a pseudo-randomized order to ensure that all 10 letters had been presented once before being repeated.

##### 3.1.3.2 Non-sign repetition

One hundred non-sign video clips were chosen from LSE-Sign: a lexical database for Spanish Sign Language (Gutierrez-Sigut, Costello, Baus, & Carreiras, 2015). Video clips for non-signs in this database were recorded by varying one phonological parameter from an existing Spanish sign language sign. The resulting non-sign kept the visual complexity of a real sign, including non-manual features. The selected non-signs contained highly perceivable handshapes and maintained the variability and complexity of locations and movements found in real signs. In order to further remove the linguistic component, and therefore ensure that the stimuli would not have any meaning for non-signers, the selected non-signs had been previously rated as non-iconic by signers of LSE (see Gutierrez-Sigut, Costello, et al., 2015 for further details). Videos were short clips of non-signs produced in a carrier sentence. They were edited to start and finish in the first and last hold of the item (see Gutierrez-Sigut, Costello, et al., 2015). A mixture of 2-handed and 1-handed signs were presented. The proportions of each type of non-sign were established to reflect the movements of the signing participants in Experiment 1: 56 non-signs were one-handed signs, 21 were right hand dominant and 23 were two-handed, with symmetrical movement of both hands. The block consisted of these 100 non-signs presented in a randomized order. Approximately 7 non-signs were presented during each of the 14 experimental trials. In Experiment 1, signers produced an average of 6 signs per trial. Non-signers were required to repeat slightly more than this number in order to encourage engagement with the task since we have previously shown that decreasing the stimulus presentation rate results in weaker LIs with the same stimuli (Payne et al., 2015).

#### 3.1.4 Procedure

Ethical approval for the study was obtained from the UCL Research Ethics Committee. All participants gave written informed consent prior to the study. The whole session, including set up time, lasted approximately 2 h.

##### 3.1.4.1 Phonological fluency block

The procedure was the same as in Experiment 1 English phonological fluency except that a blank screen of 5 s was included at the beginning of the block to ensure that baseline measures for the first trial would be calculated from resting level.

##### 3.1.4.2 Non-sign repetition block

Each trial began with a four seconds preparation period during which ‘Clear your mind’ was displayed on the screen in English. Then during the 17 s active period multiple short video clips were displayed. Following presentation of the first video clip the last frame remained on the screen for 1300 ms. During this period the participant repeated the observed non-sign. Then the next video clip appeared followed by 1300 ms to allow repetition, this sequence continued until the end of the 17 s (approximately 7 videos). Participants were instructed to repeat the non-signs with the same hand as the model, and they performed at least one example of each category copying the experimenter’s movements before the tasks. A trigger was sent to the acquisition computer when the first video clip was displayed. A ‘relax’ prompt in English then appeared for 12.5 s. As in the phonological fluency block, the first trial was preceded by a 5 s blank screen. All participants were monitored carefully during the experiment to ensure they complied with the instructions. A blank screen of 5 s was also included at the beginning of the block.

#### 3.1.5 fTCD recording and processing

For comparability between the experiments fTCD signal processing was the same as in Experiment 1, except that data from 14 instead of 19 trials were collected for each block. Visual inspection of individual trials for each participant established that the maximum left–right differences were within the selected POI. The first trial of each block was not removed, as a period of 5 s of a blank screen was included before the first trial.

### 3.2 Results

#### 3.2.1 fTCD data reliability

Due to insonation difficulties and occasional hits of the ultrasound probes while participants repeated a movement close to the head, we were not able to collect sufficient data from two participants. All participants had over eight valid epochs in all conditions; the average number of epochs for phonological fluency was 10.6 (SD = 1.9, min = 8, max = 14) and for non-sign repetition was 13.4 (SD = .63, min = 12, max = 14). Good reliability was found for the fTCD data. Split half reliability analyses demonstrated that odd and even epochs were correlated within both the phonological fluency (*r* = .81, *p* < .0001) and non-sign repetition (*r* = .62, *p* < .05) tasks.

#### 3.2.2 Mean LI and percentage of subjects left lateralized

One-sample *t*-test showed that phonological fluency was left lateralized at the group level (see Table 3 and Fig. 2, right panel). Of the 14 participants, one was right lateralized and another two, although having positive LIs, were considered low lateralized. For the non-sign repetition condition, group results were more variable. One-sample *t*-test showed that non-sign repetition LIs were not significantly different to zero and cannot be considered lateralized at a group level (see Table 3 and Fig. 2, right panel). At the individual level five participants were left lateralized, five right lateralized and four were considered low lateralized.

#### 3.2.3 LI differences between phonological fluency and non-sign repetition

A paired sample *t*-test comparing phonological fluency and non-sign repetition (mean 2.58 vs. 0.25) for all participants showed no significant difference in LI (*t*(13) = 1.7, *p* > .1). However, when we performed the same analysis after removing the participant who was strongly right lateralized for phonological fluency, there was a significant effect of condition: LIs were more positive for phonological fluency than for hand movement (mean 3.5 vs. 0.2 [*t*(12) = 3.31, *p* < .01]).

#### 3.2.4 Relationship LI and number of items generated during phonological fluency

**Fig. 4:**
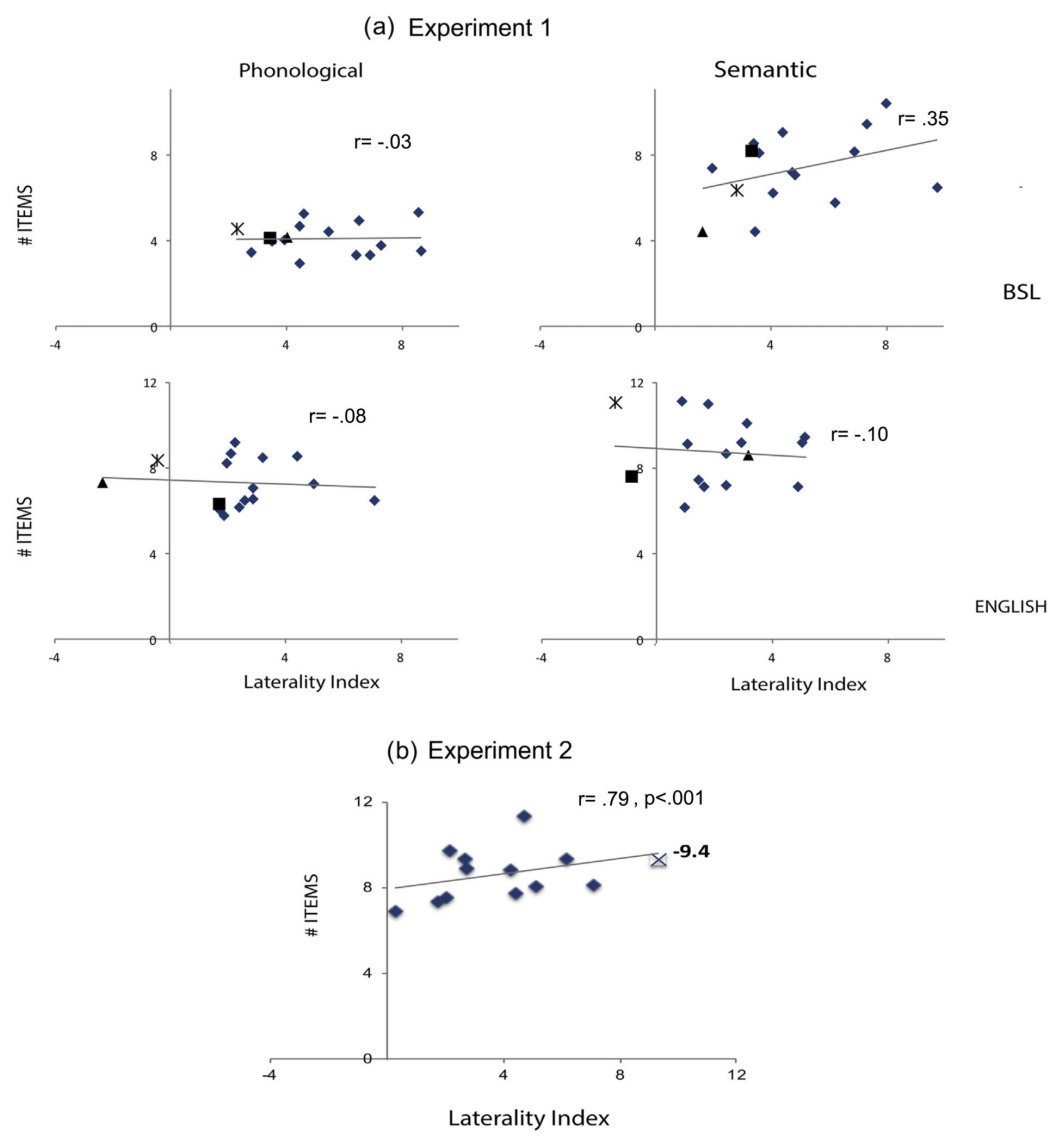
Scatterplots showing relationships between LIs and number of items produced for Experiment 1 (a) and Experiment 2 (b). For Experiment 1 the left side panel show the phonological fluency task in BSL (top) and English (bottom). The right side panel shows the semantic fluency task for BSL (top) and English (bottom). The 3 participants that had an LI lower than 0 in any of the conditions are shape coded. None of these relationships was significant. For Experiment 2 (b) the scatterplot shows the relationship between LI and number of words produced in the English phonological fluency. For the right lateralized participant the absolute value LI is plotted (X shape). The relationship was significant both when the right lateralized participant was excluded and when the absolute value LI was considered.

**Fig. 5:**
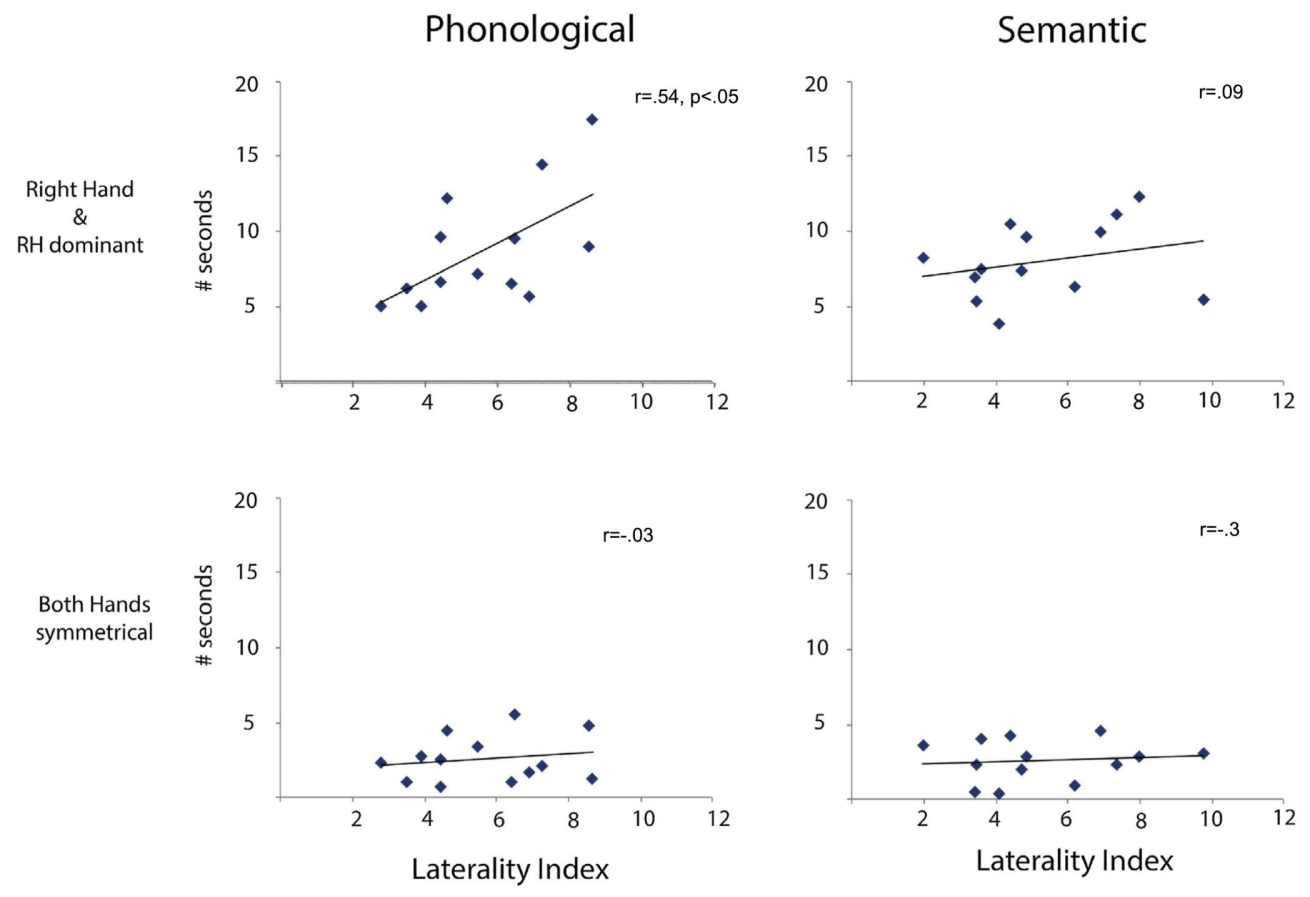
Scatterplots showing relationships between LIs and types of hand movement for the BSL phonological (left) and semantic (right) fluency tasks. The top panel show the relationships between number of seconds per trial spent in right hand only and right hand dominant movements. The bottom panel shows the relationships between number of seconds per trial spent on two-handed symmetrical movements. Only the relationship between right hand movements and phonological tasks (top left) was significant.

Strength of LI correlated with number of items produced during phonological fluency when the participant who was strongly right lateralized was excluded (*r* = .71, *p* < .05) and also when this participant was included but their *absolute* LI value was included in the analyses (*r* = .79, *p* > .001) – (see Fig. 4b). This result contrasts with the lack of correlation for hearing native signers found for the phonological fluency in English in Experiment 1. However, it is in accordance with previous results from our group from English monolinguals (Gutierrez-Sigut, Payne, et al., 2015).

#### 3.2.5 Contrasting Experiment 1 and 2: The influence of producing signs on hemispheric lateralization in signers and non-signers

**Fig. 6:**
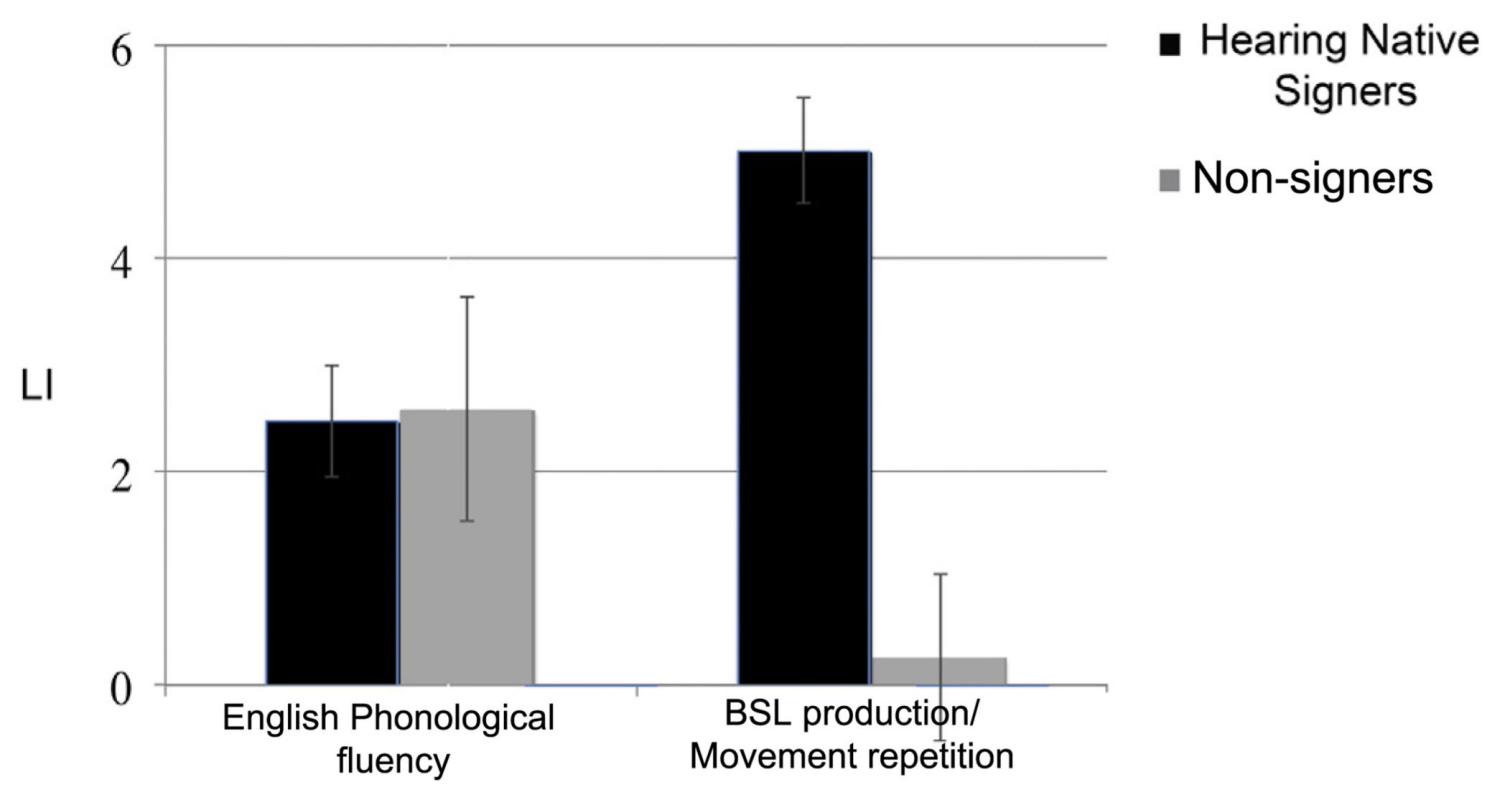
Mean LI summaries for English phonological fluency, BSL generation and non-sign repetition for signers (black) and non-signers (grey). Error bars represent standard error.

To address directly the influence of sign production on hemispheric lateralization in those for whom sign language is meaningful and those for whom it is not, we directly contrasted the strength of LI in hearing signers producing signs (during phonological and semantic fluency combined – Experiment 1) and hearing non-signers producing matched hand movements (Experiment 2). Left hemisphere lateralization was significantly stronger in signers than non-signers (*t*(28) = 5.3, *p* < .0001). Importantly, this group difference was not a general effect (see Fig. 6). The hearing signing and non-signing participants did not differ in strength of LI during the English phonological fluency task (*t*(28) = −.104, *p* > .1).

## 4 General discussion

Our aim was to investigate hemispheric lateralization during speech and sign generation in hearing native signers of BSL. Results from Experiment 1 revealed stronger left lateralization for sign than speech generation in hearing native signers. The amount of right hand movement performed during the BSL generation tasks did not correlate with the strength of LI for the semantic task and only very moderately correlated in the phonological BSL task. In Experiment 2, we demonstrated that enhanced LIs for BSL production in signers could not be attributed to activation due to hand movement alone. There was no clear pattern of left lateralization in hearing non-signing participants who performed a non-sign repetition task. Finally, no differences in strength of lateralization were found between phonological and semantic fluency tasks in either BSL or English in hearing native signers. However, relationships with behavioural measures suggest that semantic fluency, rather than phonological fluency, might be a more appropriate task to assess lateralization in BSL.

### 4.1 BSL vs. English generation

Our finding of strong left lateralization during sign generation provides additional support to the increasing body of research showing an amodal left hemisphere language processing network (Bellugi et al., 1988; Corina, 1999; Corina et al., 1999; Damasio et al., 1986; MacSweeney, Capek, et al., 2008; MacSweeney, Waters, et al., 2008). Although the spatial resolution of fTCD does not allow us to make claims about specific areas involved in BSL production, the neuroimaging literature has shown increased activity in the left parietal cortex associated with sign production. Emmorey et al. (2007) found increased left parietal activation when deaf signers named pictures in ASL than when speakers named similar pictures in English. This increased brain activation in signers has been linked to binding of phonological properties of signs both in sign production (Corina et al., 2003) and comprehension (MacSweeney, Waters, et al., 2008; MacSweeney et al., 2002). Increased left parietal activity for sign production has been also linked to the more extensive use of somatosensory and tactile feedback and the need for increased proprioceptive monitoring for sign production. In a study of spontaneous sign production in hearing native signers, who performed the task both in English and ASL, Braun et al. (2001) found increased activity for ASL production in widespread parietal somatosensory areas. Emmorey et al. (2014) studied single sign generation during picture naming in hearing native signers. Direct contrasts of ASL and English production revealed greater left parietal activation for ASL production related to phonological encoding specific to signs, somatosensory feedback and production of motor movements of the upper limbs. Accordingly, the stronger left lateralization for BSL than English generation in the current study could be accounted for by modality-dependent factors such as the greater reliance of sign language production on somatosensory feedback and a phonological encoding process that requires the selection of a handshape, a body location and a hand movement simultaneously.

A second notable finding of our study is that the stronger left lateralization found during BSL productions is not driven by motor activation alone. First, LIs during BSL production were not strongly correlated with hand movement during both generation tasks (Experiment 1). Second, non-signers did not show a clear pattern of lateralization at the group level during non-sign repetition (Experiment 2). The fact that there is not a clear pattern of lateralization for the non-signers suggests that the higher LIs, found for signers during BSL production, are linked to language factors (e.g. sign phonological encoding, linguistic somatosensory feedback and motor planning) rather than to more general motor activation and somatosensory feedback mechanisms, which are common to both BSL generation and non-sign repetition. Our results are in line with an fMRI study by Corina et al. (2003) who found that in right handed signing participants, activation for sign production was left lateralized regardless of the hand used to produce the signs, suggesting that the linguistic motor programming of both hands is driven by the same left hemisphere regions.

The lack of a consistent pattern of lateralization for the non-signers is surprising. Although, non-sign repetition by non-signers involves cognitive processes different to those needed to spontaneously generate language, nevertheless, to perform the task accurately, participants were required to track the shape, position and movement of the hands. Lack of expertise in hand movements is unlikely to account for this pattern since they were all equally inexperienced in signing but it was not the case that all participants showed weak lateralization. Rather, one third were significantly left lateralized, one third were significantly right lateralized and one third were low lateralized. Non-signers might have approached the non-sign repetition task differently. One possibility is that some of the non-signers treated the non-sign repetition as a visuospatial receptive task, increasing the involvement of the right hemisphere, as has been shown in previous fTCD studies of visuo-spatial processing (e.g., Payne et al., 2015; Rosch, Bishop, & Badcock, 2012; Whitehouse, Badcock, Groen, & Bishop, 2009). Another possible explanation for the variability within the non-signing group is that, although the non-signs used in the current study had been rated as non-iconic (Gutierrez-Sigut, Costello, et al., 2015) and the instructions emphasized that they had no meaning, it is possible that some participants sought meaning in the signs, and therefore increased the involvement of the left hemisphere. Finally, it is possible that the fTCD signal was weaker in some of the non-signers because they were performing a repetition task rather than the generation task performed by signers. Generation tasks tend to result in stronger left lateralized LIs than receptive tasks (Badcock, Nye, et al., 2012; Buchinger et al., 2000; Stroobant, Buijs, & Vingerhoets, 2009). Stroobant et al. (2009) showed that reading aloud fragments of natural text resulted in weaker left lateralization than sentence construction (from words in a random order) or phonological fluency. Further research into movement generation is necessary to fully address this issue.

In summary, although it is likely that non-signers were using different strategies to perform the non-sign repetition task to those used by signers in the sign generation task, nevertheless the movements they produced matched the amount of hand movement produced, on average, by signers during the sign generation task. If the motoric planning or a general mechanism to track the position and movements of the hands and arms was strongly influencing the LIs, a general trend towards left lateralization should have been more evident.

Consistent with our previous results in spoken English (Gutierrez-Sigut, Payne, et al., 2015), the present results support the idea that the blood velocity changes, as measured by fTCD, are not predominantly driven by motor processes but by more linguistic processes. This is the first study to show that fTCD measures of language lateralization are robust to movement even during production of a signed language, which requires constant and mostly asymmetric, movement of the hands. Indeed, the use of fTCD allows language lateralization to be assessed in a more naturalistic experimental situation where participants can produce the complete movement of the signs and use both hands without restrictions. The fact that fTCD reliably measures lateralization during natural language production makes it more feasible to include young signers and those with cochlear implants in experimental groups. This would broaden our understanding of a very heterogeneous population.

### 4.2 Phonological vs. semantic fluency

With regard to lateralization in different language sub-domains, we found the expected similar levels of lateralization between phonological and semantic tasks in English. LIs between phonological and semantic generation were also similar in BSL. This result suggests that the fTCD signal is affected similarly by linguistic and cognitive processes involved in language generation in both modalities. In accordance with previous behavioural studies in English (Crowe, 1998; Hurks et al., 2006; Monsch et al., 1994) and BSL (Marshall et al., 2014), participants produced more items during the semantic than the phonological fluency in both languages.

It is worth noting that the English and BSL phonological tasks are not equivalent. In BSL the stimulus is a handshape (a phonological parameter of signs). In contrast, in English the stimulus is a letter and is therefore an orthographic/visual representation of the target phoneme, rather than an auditory cue which would be directly analogous to the pictured handshape cue in the BSL task. These different task demands did not result in a significant difference of LIs between the phonological and semantic condition in either language. However, subtle differences in performance on the BSL phonological and semantic fluency tasks are worth noting. First, in the BSL phonological, but not semantic, task we found a moderate, but significant, correlation between the LI and the movement of the right hand. Inspection of the videos showed a motoric rehearsing strategy in the BSL phonological task. Participants tended to hold the cued handshape in their right hand. They would then move the hand to different locations where they rehearsed several movements, thus increasing the amount of movement that was coded, yet only occasionally recovering some extra signs. Second, the BSL phonological fluency task is more difficult than the semantic fluency task (see Marshall et al., 2014 for discussion). Although native signers show good phonological awareness for sign language when tested on explicit judgement tasks (e.g., Corina, Hafer, & Welch, 2014; MacSweeney, Capek, et al., 2008; MacSweeney, Waters, et al., 2008) it is likely that signers are not very familiar with the nature of the phonological fluency task tested here. For example, I-spy games, based on initial phonemes of words, are common children’s games in spoken but not signed languages.

Phonological fluency is traditionally considered the ‘‘gold standard task” for assessing lateralization during speech production with fTCD. Consistent with our previous findings in English (Gutierrez-Sigut, Payne, et al., 2015), the current results show that both phonological and semantic fluency can be reliable tasks for assessing language lateralization with fTCD in speech production. For sign language production however, there are concerns that non-linguistic factors such us increased task difficulty or the use of a motoric rehearsing strategy might be contributing to the lateralization strength in the BSL phonological task. Semantic fluency may be more appropriate when investigating language lateralization during BSL production since it is directly comparable to a similar task in English, more intuitive to perform and is not compromised by the accompanying “non-linguistic, searching” hand movements that participants tend to make during the phonological task.

### 4.3 Effects of language background

Both hearing signers and non-signers showed similar LIs during English phonological fluency, suggesting that knowing a sign language does not affect lateralization strength during English production as measured by fTCD. However, in line with our previous results in English monolinguals (Gutierrez-Sigut, Payne, et al., 2015) the amount of words produced by non-signers during the phonological fluency task correlated with the strength of laterality. We did not find this correlation for the hearing native signers for words or for signs. Very few studies have reported the relationship or lack of relationship between amount or quality of language produced and the strength of the fCTD signal (see Gutierrez-Sigut, Payne, et al., 2015 for discussion). Further research is needed to examine this issue and to address the possibility raised by the current pattern of results which suggest that bilingualism may influence this relationship.

## 5 Conclusions

By examining language lateralization using fTCD with a task other than the ‘gold standard’ phonological fluency task, we have shown that semantic fluency may in fact be more appropriate for assessing language lateralization in signed languages. We found evidence of stronger left lateralization for BSL than for English production, across both semantic and phonological fluency tasks. Importantly, we showed that this increased lateralization cannot be attributed to motoric activity alone. Although fTCD methodology is more basic than other neuroimaging techniques, simultaneous measurement of fully articulated behavioural responses allows for correlational analyses that can shed light on the factors affecting lateralization patterns in different language modalities. Further studies are required to determine what cognitive and linguistic processes contribute to this enhanced left lateralization for sign language production.

## Figures and Tables

**Table 1:** The left side of the table shows the mean LI values and group one-sample *t*-tests for each condition in Experiment 1. The right side of the table shows the number (percentage between brackets) of participants left, right and ‘low’ lateralized in each condition.


Language	Task	LI				Left lateralized	Right lateralized	Low laterality
		Mean	SD	*t*	*p*	#(%)	#(%)	#(%)

English	Phonological	2.5	2.1	4.7	<.0001	13 (81.5%)	1 (6%)	2 (12.5%)
	Semantic	2.2	1.9	4.6	<.0001	10 (63%)	1 (6%)	5 (31%)
BSL	Phonological	5.2	1.9	10.6	<.0001	16 (100%)	0	0
	Semantic	4.8	2.3	8.4	<.0001	14 (87.5%)	0	2 (12.5%)


**Table 2:** Descriptive statistics for the number of correct items generated in each condition in Experiments 1 and 2.

Language	Language task	Mean number of items per trial	SD	Minimum	Maximum
*Experiment 1*					
English	Phonological	7.3	1.1	2	13
	Semantic	8.7	1.6	4	16
BSL	Phonological	4.1	.7	2	5
	Semantic	7.3	1.7	4	10
*Experiment 2*					
English	Phonological	8.6	1.2	2	14

**Table 3:** The left side of the table shows the mean LI values and group one-sample *t*-tests for each condition in Experiment 2. The right side of the table shows the number (percentage between brackets) of participants left, right and ‘low’ lateralized in each condition.


Task	LI				Left lateralized	Right lateralized	Low laterality	Mean number of items per trial
	Mean	SD	*t*	*p*	# (%)	# (%)	# (%)	

Phonological fluency	2.58	3.9	2.47	<.05	11 (79%)	1 (7%)	2 (14%)	8.6
Non-sign repetition	0.25	2.9	0.3	>.1	5 (35.7%)	4 (28.6%)	5 (35.7%)	


## Footnotes

1 Note that in this sign type the non-dominant hand usually adopts an unmarked handshape, e.g. B-flat and serves as a basis for the dominant hand movement. The movement of the non-dominant hand therefore is very limited for these instances.

2 The number of trials was reduced with respect to Experiment 1 to reduce participants fatigue and promote engagement with the task.
